# Ambient PM_2.5_, O_3_, and NO_2_ Exposures and Associations with Mortality over 16 Years of Follow-Up in the Canadian Census Health and Environment Cohort (CanCHEC)

**DOI:** 10.1289/ehp.1409276

**Published:** 2015-11-01

**Authors:** Dan L. Crouse, Paul A. Peters, Perry Hystad, Jeffrey R. Brook, Aaron van Donkelaar, Randall V. Martin, Paul J. Villeneuve, Michael Jerrett, Mark S. Goldberg, C. Arden Pope, Michael Brauer, Robert D. Brook, Alain Robichaud, Richard Menard, Richard T. Burnett

**Affiliations:** 1 Environmental Health Science and Research Bureau, Health Canada, Ottawa, Ontario, Canada; 2 Department of Sociology, University of New Brunswick, Fredericton, New Brunswick, Canada; 3 College of Public Health and Human Sciences, Oregon State University, Corvallis, Oregon, USA; 4 Air Quality Research Division, Environment Canada, Downsview, Ontario, Canada; 5 Dalla Lana School of Public Health, University of Toronto, Toronto, Ontario, Canada; 6 Department of Physics and Atmospheric Science, Dalhousie University, Halifax, Nova Scotia, Canada; 7 Institute of Health: Science, Technology and Policy, Carleton University, Ottawa, Ontario, Canada; 8 School of Public Health, University of California, Berkeley, Berkeley, California, USA; 9 Department of Medicine, McGill University, Montreal, Quebec, Canada; 10 Division of Clinical Epidemiology, Research Institute of the McGill University Hospital Centre, Montreal, Quebec, Canada; 11 Department of Economics, Brigham Young University, Provo, Utah, USA; 12 School of Population and Public Health, University of British Columbia, Vancouver, British Columbia, Canada; 13 Division of Cardiovascular Medicine, University of Michigan, Ann Arbor, Michigan, USA; 14 Atmospheric Science and Technology Directorate, Environment Canada, Dorval, Quebec, Canada

## Abstract

**Background:**

Few studies examining the associations between long-term exposure to ambient air pollution and mortality have considered multiple pollutants when assessing changes in exposure due to residential mobility during follow-up.

**Objective:**

We investigated associations between cause-specific mortality and ambient concentrations of fine particulate matter (≤ 2.5 μm; PM_2.5_), ozone (O_3_), and nitrogen dioxide (NO_2_) in a national cohort of about 2.5 million Canadians.

**Methods:**

We assigned estimates of annual concentrations of these pollutants to the residential postal codes of subjects for each year during 16 years of follow-up. Historical tax data allowed us to track subjects’ residential postal code annually. We estimated hazard ratios (HRs) for each pollutant separately and adjusted for the other pollutants. We also estimated the product of the three HRs as a measure of the cumulative association with mortality for several causes of death for an increment of the mean minus the 5th percentile of each pollutant: 5.0 μg/m^3^ for PM_2.5_, 9.5 ppb for O_3_, and 8.1 ppb for NO_2_.

**Results:**

PM_2.5_, O_3_, and NO_2_ were associated with nonaccidental and cause-specific mortality in single-pollutant models. Exposure to PM_2.5_ alone was not sufficient to fully characterize the toxicity of the atmospheric mix or to fully explain the risk of mortality associated with exposure to ambient pollution. Assuming additive associations, the estimated HR for nonaccidental mortality corresponding to a change in exposure from the mean to the 5th percentile for all three pollutants together was 1.075 (95% CI: 1.067, 1.084). Accounting for residential mobility had only a limited impact on the association between mortality and PM_2.5_ and O_3_, but increased associations with NO_2_.

**Conclusions:**

In this large, national-level cohort, we found positive associations between several common causes of death and exposure to PM_2.5_, O_3_, and NO_2_.

**Citation:**

Crouse DL, Peters PA, Hystad P, Brook JR, van Donkelaar A, Martin RV, Villeneuve PJ, Jerrett M, Goldberg MS, Pope CA III, Brauer M, Brook RD, Robichaud A, Menard R, Burnett RT. 2015. Ambient PM_2.5_, O_3_, and NO_2_ exposures and associations with mortality over 16 years of follow-up in the Canadian Census Health and Environment Cohort (CanCHEC). Environ Health Perspect 123:1180–1186; http://dx.doi.org/10.1289/ehp.1409276

## Introduction

It is challenging to estimate associations between long-term exposure to air pollution and health outcomes because pollution levels vary both in space and time. Exposure assessment is further complicated due to people moving several times throughout their lifetimes. In Canada, for example, nearly half of all individuals moved at least once within the 5-year period between 2001 and 2006 (Statistics Canada 2006). Many longitudinal cohorts include location information for subjects only at study inception. Estimates of exposure are therefore often assigned to persons’ location at baseline as a marker for long-term exposure (Beelen et al. 2008; Jerrett et al. 2009a, 2009b; Pope et al. 2002), which, because of residential mobility patterns, inherently leads to exposure misclassification.

Despite these limitations and challenges, several large cohort studies based in the United States (Jerrett et al. 2013; Krewski et al. 2009; Laden et al. 2006), Europe (Beelen et al. 2008; Carey et al. 2013; Cesaroni et al. 2013; Raaschou-Nielsen et al. 2012), and Canada (Crouse et al. 2012) have demonstrated robust and relatively consistent associations between long-term exposures to ambient pollution and risk of mortality from nonaccidental causes, cardiovascular diseases, and lung cancer. In our original analysis of the national, population-based Canadian Census Health and Environment Cohort (CanCHEC) (Crouse et al. 2012), we assigned estimates of ambient concentrations of fine particulate matter [particulate matter ≤ 2.5 μm in aerodynamic diameter (PM_2.5_)] to the enumeration area of residence (enumeration areas range in size from approximately 650 dwellings in urban areas to < 100 dwellings in rural areas) at baseline to 2.1 million nonimmigrant adults across Canada who completed the 1991 long-form census. We reported hazard ratios (HRs) for mortality from nonaccidental causes and cardiovascular disease of 1.10 [95% confidence intervals (CIs): 1.05, 1.15] and 1.15 (95% CI: 1.07, 1.24), respectively, per an increase of 10 μg/m^3^ in PM_2.5_. These findings were within the ranges of associations reported in other cohort studies (Cesaroni et al. 2013; Krewski et al. 2009; Laden et al. 2006).

Given our acquisition of additional and enhanced data sets, the objective of the present study is to present an extensive analysis of the associations between cause-specific mortality and long-term exposures to three ambient pollutants among subjects in the CanCHEC. We build on our previous work by adding five additional years of follow-up, tracking subjects’ annual residential histories for the purpose of assigning time-varying exposures, including immigrants, including subjects living in the far north, using improved estimates of satellite-derived PM_2.5_, adding exposure estimates for ozone (O_3_) and nitrogen dioxide (NO_2_), and assigning more spatially refined exposure assessments by using subjects’ 6-digit residential postal code instead of the larger enumeration area of residence.

## Methods

*The study cohort.* CanCHEC has been described in detail previously (Crouse et al. 2012; Peters et al. 2013). Briefly, it is a population-based cohort of subjects who were ≥ 25 years of age at baseline; a usual resident of Canada on the census reference day (4 June 1991); not a resident of an institution such as a prison, hospital, or nursing home; and among the 20% of Canadian households (~ 3.6 million respondents) selected for enumeration with the long-form census questionnaire. Subjects in CanCHEC were linked to the Canadian Mortality Database using deterministic and probabilistic linkage methods from 4 June 1991 through 31 December 2006 (Peters et al. 2013). The date of death and the underlying cause of death were extracted from death certificates coded by nosologists to the *International Classification of Diseases, 9th Revision* (ICD-9) for deaths before 2000, and to the *10th Revision* (ICD-10) for those that occurred from 2000 onward. The original cohort included the enumeration area of residence in 1991 and mortality follow-up through 2001. We extended the years of follow-up through 2006 and linked annual place of residence (6-digit postal code) using Historical Tax Summary Files from 1984 through 2006 (Peters et al. 2013). In urban areas, 6-digit residential postal codes most often correspond to one side of a city block or to a single apartment building; in rural areas a single postal code may represent a larger area.

*Assignment of exposure to ambient air pollution.* We assigned estimates of concentrations to PM_2.5_, O_3_, and NO_2_ to the representative point of each subject’s residential postal code for every available year between 1984 and 2006 (i.e., up to 7 years before baseline). A subject’s exposure was coded as missing in years for which no residential postal code was available, which could indicate that the subject had left Canada, moved to an institution, or had not filed an income tax return that year.

Our estimates of PM_2.5_ were derived from observations from three satellite instruments [MISR (Multi-angle Imaging SpectroRadiometer; https://www-misr.jpl.nasa.gov), MODIS (Moderate Resolution Imaging Spectroradiometer; http://modis.gsfc.nasa.gov), and SeaWiFS (Sea-viewing Wide Field-of-view Sensor; http://oceancolor.gsfc.nasa.gov/SeaWiFS/)] to represent median annual concentrations during the period 1998–2006 (van Donkelaar et al. 2015) for each grid cell. The PM_2.5_ estimates were available on a grid with a spatial resolution of approximately 10 km × 10 km and included coverage up to 70°N. These estimates combined the values used in our previous study (van Donkelaar et al. 2010) with optimal estimation-based values (van Donkelaar et al. 2013) to produce an improved representation of PM_2.5_ with extended temporal range and greater accuracy. Temporal variation in PM_2.5_ between 1998 and 2006 was inferred from two radiometrically stable satellite instruments (MISR and SeaWiFS) (Boys et al. 2014).

We generated an O_3_ surface representing the average of the daily 8-hr maximum concentrations in the warm seasons (1 May–31 October) for the period 2002–2009 across Canada and the United States with 21-km horizontal resolution through an optimal interpolation technique adapted to air pollutants (Robichaud and Ménard 2014). This method linearly combines the hourly modeled O_3_ surface from Canadian air quality forecast models with observations available in both countries [i.e., U.S. Environmental Protection Agency AIRNow observations (http://www.airnow.gov)]. The weights attributed to the model output and observations were optimized using an established method of data assimilation (Kalnay 2003). The modeled O_3_ surface was provided by the CHRONOS (Canadian and Hemispheric Regional Ozone and NO_x_ System) operational regional air quality forecast model (Pudykiewicz et al. 1997). This blending, or data fusion, provides more physically realistic estimates of ambient O_3_ concentrations over areas lacking monitoring data compared to standard interpolation techniques. Method validation was carried out by reprocessing the objective analysis with a reduced data set (90%). Therefore, the remaining (10%) is used for cross-validation. The objective analysis (data fusion of model and observations) correctly estimated within a factor of two the missing data 64–97% of the time, and significantly reduced both systematic and random errors in the estimates relative to the model alone (Robichaud and Ménard 2014). The 21-km grid values for this metric were then assigned to the 6-digit postal codes of the subjects in our cohort.

We estimated residential exposures to 2006 annual mean concentrations of NO_2_ using a national land use regression model (LUR) developed from National Air Pollution Surveillance (NAPS; http://www.ec.gc.ca/rnspa-naps/) monitoring data using methods reported by Hystad et al. (2011). The updated LUR model applied here includes 2005–2011 satellite NO_2_ estimates (Lamsal et al. 2008), road length within 10 km, area of industrial land use within 2 km, and mean summer rainfall. This model explained 73% of the variation in 2006 NAPS measurements with a root mean square error of 2.9 parts per billion. The model also predicted 43% of the variability in NO_2_ measurements collected in seven cities (*n* = 35–196 sites per city; Hystad et al. 2011) based on special monitoring campaigns conducted to develop land use models. To capture fine-scale variation in vehicle emissions, kernel density measures (i.e., smoothed surfaces describing densities of roadways) were applied as multipliers to the LUR model for highways (increasing LUR concentrations 65% for the top 10th percentile of measures at the edge of highways decreasing linearly to 0% at 300 m from a highway) and for major roads (increasing LUR concentrations 20% for the top 10th percentile of measures at the edge of major roads decreasing linearly to 0% at 100 m from a major road). This method allowed us to capture complex patterns in roadway emissions (e.g., the influence of multiple roadways, intersections, off-ramps).

For each year of follow-up we estimated for each subject a 7-year moving window of past concentrations to each pollutant—with a single-year lag—beginning with data from 1984 (i.e., the earliest year of data available to us, and thus the largest window of exposure available) providing that exposures (i.e., postal codes) were available in at least 4 of the 7 years. For example, a subject’s moving window of exposure for 1991 would be estimated as the mean of the exposures assigned to that subject’s postal code over the 7 years 1984–1990. If postal code information was missing in > 3 of those 7 years, no moving window of exposure would be assigned, and the subject would be excluded from analysis in that year. This moving window of exposure allowed us to incorporate into our models the variability in exposures associated with annual residential mobility patterns. This method also allowed us to retain subjects for whom we had incomplete postal code histories, rather than censoring them in years for which we had no locational information. The spatial structure of each exposure surface was not assessed in a time-dependent manner, and thus variation in exposure for each subject was attributed solely to residential mobility. There are insufficient historical observations of these pollutants to describe the long-term spatial and temporal patterns across all of Canada for this period (i.e., fixed-site stations are located only in large cities and have incomplete historical records). We did, however, compare our satellite-derived estimates of PM_2.5_ with observations from fixed-site stations in 10 cities for which long-term data were available (see Supplemental Material, Figure S1). Here, we found a Pearson correlation of 0.90 between the estimates used in our analyses (i.e., median concentrations 1998–2006) and long-term means calculated with observations from 1984 through 2006.

*Main statistical analyses.* We used Cox proportional hazards models to estimate the associations between air pollution and mortality. We estimated HRs stratified by sex and by 5-year age groups from ages 25 to 89 years. We restricted our study to subjects < 90 years of age due to potential inaccuracies in record linkages among older subjects (e.g., the address reported on the annual income tax filings of older subjects may reflect those of next of kin, or of institutional facilities). Subjects were censored at time of death or if they were lost to follow-up due to end of study period or lack of postal code information.

We adjusted our models for aboriginal ancestry, visible minority status, immigrant status, marital status, highest level of education, employment status, occupational classification, and quintiles of household income (see Supplemental Material, Table S1, for coding). We also calculated time-varying contextual variables from the closest census year (i.e., either 1991, 1996, 2001, or 2006) adjusted for regional variations across Canada (i.e., census division means subtracted from census-tract means) describing the proportion of unemployed adults, the proportion of adults who had not completed high school, and the proportion of individuals in the lowest income quintile. Census tracts correspond roughly to the size of a neighborhood, and census divisions correspond roughly to the size of a city or county. These time-varying contextual covariates were reassigned each year, taking into account each subject’s current residential location during each year of follow-up.

We developed models for nine causes of mortality including all nonaccidental causes, lung cancer, cardiometabolic diseases (i.e., circulatory plus diabetes), diabetes, cardiovascular diseases, ischemic heart disease, cerebrovascular disease, diseases of the respiratory system, and chronic obstructive pulmonary disease and allied conditions (chronic obstructive pulmonary disease; COPD). Table 1 presents specific ICD-9 and ICD-10 codes for each cause of death.

**Table 1:** Hazard ratios (95% CIs) for mortality by pollutant in single- and multi-pollutant models: models stratified by age and sex, adjusted for personal*^a^* and contextual*^b^* covariates.

Model	Nonaccidental^*c*^ (301,115)	Trachea, bronchus, and lung cancers^*d*^ (30,545)	Cardiometabolic diseases^*e*^ (117,495)	Diabetes^*f*^ (9,330)	Cardiovascular^*g*^ (98,970)	Ischemic heart disease^*h*^ (63,050)	Cerebrovascular^*i*^ (19,725)	Diseases of the respiratory system^*j*^ (24,900)	COPD and allied conditions^*k*^ (14,170)
PM_2.5_ alone	1.035 (1.029, 1.041)	1.031 (1.013, 1.049)	1.038 (1.029, 1.047)	1.149 (1.113, 1.186)	1.030 (1.021, 1.040)	1.085 (1.073, 1.099)	0.960 (0.939, 0.980)	0.973 (0.955, 0.992)	0.989 (0.964, 1.063)
PM_2.5_ adjusted for O_3_ and NO_2_	1.011 (1.003, 1.020)	1.038 (1.011, 1.066)	0.998 (0.985, 1.011)	1.060 (1.011, 1.112)	0.994 (0.979, 1.008)	1.027 (1.008, 1.046)	0.938 (0.908, 0.969)	0.978 (0.950, 1.007)	1.005 (0.967, 1.004)
O_3_ alone	1.031 (1.026, 1.036)	1.006 (0.990, 1.023)	1.046 (1.037, 1.054)	1.156 (1.121, 1.190)	1.037 (1.028, 1.047)	1.087 (1.075, 1.100)	0.981 (0.961, 1.001)	0.971 (0.953, 0.989)	0.972 (0.949, 0.996)
O_3_ adjusted for PM_2.5_ and NO_2_	1.018 (1.010, 1.026)	0.973 (0.950, 0.997)	1.043 (1.031, 1.056)	1.110 (1.063, 1.160)	1.038 (1.024, 1.052)	1.062 (1.045, 1.080)	1.023 (0993, 1.055)	0.981 (0.955, 1.007)	0.961 (0.928, 0.996)
NO_2_ alone	1.052 (1.045, 1.059)	1.074 (1.051, 1.097)	1.040 (1.029, 1.051)	1.039 (0.999, 1.080)	1.041 (1.028, 1.053)	1.063 (1.047, 1.079)	1.004 (0.977, 1.031)	1.036 (1.012, 1.061)	1.068 (1.035, 1.102)
NO_2_ adjusted for PM_2.5_ and O_3_	1.045 (1.037, 1.052)	1.067 (1.043, 1.091)	1.032 (1.021, 1.044)	1.003 (0.963, 1.044)	1.035 (1.022, 1.048)	1.042 (1.026, 1.058)	1.020 (0.993, 1.049)	1.048 (1.022, 1.074)	1.075 (1.040, 1.110)
Hazard ratios are per mean – 5th percentile (i.e., 5.0-μg/m^3^, 9.5-ppb, and 8.1-ppb increases in PM_2.5_, O_3_, and NO_2_, respectively); number of deaths in parentheses is rounded to nearest 5. aAboriginal ancestry, visible minority status, highest level of education, employment status, occupational class, immigrant status, marital status, income quintile. ^***b***^Census division and census tract–census division percent of immigrants, percent of adults without high school diploma, percent of subjects in lowest income quintile. ^***c***^ICD-9: < 800; ICD-10: A–R. ^***d***^ICD-9: 162; ICD-10: C33–C34. ^***e***^ICD-9: 390–459, 250; ICD-10: I00–I99, E10–E14. ^***f***^ICD-9: 250; ICD-10: E10–E14. ^***g***^ICD-9: 410–440; ICD-10: I20–I25, I30–I51, I60–I69, I70. ^***h***^ICD-9: 410–414; ICD-10: I20–I25. ^***i***^ICD-9: 430–438; ICD-10: I60–I69. ^***j***^ICD-9: 460–519 ; ICD-10: J00–J98. ^***k***^ICD-9: 490–496 ; ICD-10: J19–J46.									

We developed models with individual pollutants and with all three pollutants together (i.e., multi-pollutant models). In addition, we determined the product of the HRs for all possible pairs of pollutants and all three pollutants together based on the corresponding multiple pollutant models. We also calculated 95% CIs of the HR product using covariance of the HR estimates. We interpreted the HR associated with a given pollutant based on a survival model that includes all three pollutants together as the marginal risk, whereas we interpreted the product of the HRs based on a survival model with all three pollutants together as the cumulative risk estimate.

More specifically, we estimated cumulative risk estimates assuming additive effects of combined pollutant exposures on mortality. This method was originally developed for an earlier study (Lippmann et al. 2013), and expanded elsewhere (Jerrett et al. 2013). Essentially, the cumulative risk estimate represents the relative hazard for 1-unit increases in all three pollutants compared with that for no increase in any of the three exposures.

For this additive model, let *x*´ = (*x*_1_,…*x_p_*) represent concentrations of P air pollutants (i.e., PM_2.5_, O_3_, and NO_2_,). We denote the HR based on the combination of the P pollutants evaluated at *x* as the Cumulative Risk Index (CRI) and define it as

*CRI* = exp[Σ*^^P^^__p__* __= 1__β^ˆ^*_p_x_p_*] ≡ exp(β^ˆ^´*x*) = ∏*^^P^^__p__* __= 1__*_JHR___p__*_,_

where β^ˆ^´ = (β^ˆ^_1_,…β^ˆ^*_p_*) are the estimates of the log-hazard ratio for the P pollutants estimated in a survival model consisting of all P pollutants together, with *JHR_P_* = exp(β^ˆ^*_p_x_p_*) denoting the cumulative hazard ratio for the *p*th pollutant in a multi-pollutant survival model. Further denote *Cov*(β^ˆ^) as the variance–covariance matrix of β^ˆ^. The 95% confidence interval of CRI is defined by exp[β^ˆ^´*x* ± 1.96 × β^ˆ^*Cov*^(^β^ˆ)^β^ˆ^´].

If the pollutants are uncorrelated, we note that ∏*^^P^^__p__* __= 1__*_JHR___p___=_* ∏*^^P^^__p__* __= 1__*_IHR___p___,_* where *IHR_p_* denotes the hazard ratio of the *p*th pollutant from a survival model that contains only this pollutant, and thus the association with each pollutant is estimated independent of the other pollutants. If all *p* pollutants are positively correlated, then ∏*^^P^^__p__* __= 1__*_JHR___p___<_* ∏*^^P^^__p__* __= 1__*_IHR___p___._* That is, information in one pollutant will also be contained in the other pollutants, so one does not necessarily require all *p* pollutants to fully represent the cumulative impact on survival of the pollution mixture. Comparisons of CRIs based on selected subsets of the P pollutants can be used to interpret the associations with mortality among various pollutant subsets. Moreover, comparisons of risk estimates based on pollutants estimated cumulatively and independently provide a means of understanding the impacts of the atmospheric mixture on survival.

We calculated our HRs per increment of the mean minus the 5th percentile of exposure, namely 5.0 μg/m^3^ for PM_2.5_, 9.5 ppb for O_3_, and 8.1 ppb for NO_2_. The HR evaluated at this exposure contrast approximates the average of the HRs among all subjects based on their individual exposure to either a single pollutant or multiple pollutants, and thus represents the relative risk for the cohort as whole. We chose not use the interquartile range (IQR) for each pollutant (i.e., 5.8 μg/m^3^ for PM_2.5_, 9.9 ppb for O_3_, and 10.5 ppb for NO_2_) because we are uncertain as to whether an IQR contrast for all three pollutants examined simultaneously exists within the cohort’s exposure profile whereas the mean of each pollutant clearly does. We further limited the contrast to the mean minus the 5th percentile since setting the lower bound on the exposure contrast to zero exposure is not likely achievable and thus would unduly inflate the HR.

*Additional analyses.* We tested for effect modification by selected personal characteristics for each pollutant with nonaccidental mortality and with cardiometabolic diseases. Specifically, we used Cochran’s Q-statistic (Axelson 1980) to test heterogeneity in HRs by age during follow-up (i.e., only used follow-up of subjects during specified age range), sex, income, and immigrant status.

To evaluate the impact of tracking residential mobility on our associations, we created a “baseline exposure” cohort for which we assumed that the only address information available was postal code at baseline. Here we assumed that exposure at baseline was an adequate proxy of long-term exposures. We therefore assigned exposure estimates to subjects with available postal code information in 1991, and censored only at time of death. We developed these single-pollutant models for nonaccidental mortality only.

Two important risk factors for mortality (cigarette smoking habits and obesity) were not captured in the census. We therefore used a method (Shin et al. 2014) to mathematically adjust our HRs by examining the relationship between these missing risk factors and air pollution in an ancillary data set that did include information on smoking and obesity as well as the other variables included in our survival models. Briefly, with this method we adjusted the observed HR for a series of risk factors not reported in our data set while simultaneously controlling for risk factors that were included in our survival model (e.g., education, income, contextual variables). This method requires estimates of the multivariate linear association between the variables in the survival model and the variables we indirectly adjusted for. We obtained this association from an analysis of the national Canadian Community Health survey, for which we assigned our estimates of exposure to each subject; the details of the method are reported elsewhere (Shin et al. 2014). We made these adjustments for both the single- and multi-pollutant model HRs.

Last, we generated plots of the concentration–response curves to examine the shape of the relationship between each pollutant and nonaccidental mortality using restricted cubic spline functions with two degrees of freedom. All analyses were conducted in SAS version 9.3 (SAS Institute Inc.).

## Results

Our cohort consisted of 2,521,525 subjects at baseline who contributed to a total of 36,377,506 person-years of follow-up (see Supplemental Material, Table S1). About 19% of subjects were immigrants to Canada. At baseline, the distribution of exposures (i.e., minimum, 25th percentile, median, 75th percentile, maximum, and mean) to PM_2.5_ were 0.9, 6.0, 8.6, 11.8, 17.6, and 8.9 μg/m^3^, respectively; to O_3_ were 10.7, 34.3, 39.0, 44.2, 60, and 39.6 ppb, respectively; and to NO_2_ were 0, 6.0, 10.4, 16.5, 51.5, and 11.6 ppb, respectively. Generally, visible minorities and those reporting aboriginal ancestry tended to have lower exposures than others, and immigrants tended to have higher exposures than Canadian-born subjects. The differences in mean exposures between these groups relate to urban–rural differences in residence. See Supplemental Material, Figure S2A–C for maps of the spatial patterns of PM_2.5_, O_3_, and NO_2_ concentrations, respectively. At baseline, exposure to PM_2.5_ was correlated with exposure to O_3_ (*r* = 0.73), but less so with NO_2_ (*r* = 0.40), and the correlation between exposure to NO_2_ and O_3_ (*r* = 0.19) was smaller than that between the others (all three significant at *p* < 0.001). Approximately 301,115 subjects died during the 16 years of follow-up. Table 1 presents numbers of deaths by individual causes.

*Single-pollutant models.* We present in Table 1 the HRs and 95% CIs for the associations between cause-specific mortality and PM_2.5_, O_3_, and NO_2_. Individually, all three pollutants were positively associated with deaths from nonaccidental causes, cardiometabolic diseases, diabetes alone, and ischemic heart disease, but none were associated with deaths from cerebrovascular disease. Increases in exposure per increment of the mean – 5th percentile of each pollutant were associated with approximately 4–5% increased risk of mortality from nonaccidental causes.

*Multi-pollutant models.* Table 1 also shows results of the multi-pollutant models. Despite correlations between the pollutants, all three were associated with selected causes of mortality when adjusted for the other pollutants. In these models, PM_2.5_ was associated with elevated risk of mortality from nonaccidental causes, lung cancer, diabetes, and ischemic heart disease. O_3_ was associated with increased risk of mortality from nonaccidental causes, cardiometabolic diseases, diabetes, cardiovascular disease, ischemic heart disease, and cerebrovascular disease. NO_2_ was associated with mortality from nonaccidental causes, lung cancer, cardiometabolic diseases, cardiovascular disease, ischemic heart disease, respiratory disease, and COPD. Here we also found weaker evidence of associations with diabetes and cerebrovascular disease.

*Cumulative risk estimates.* We present the cumulative risk estimates in Table 2. The cumulative risk estimates were derived from models that were stratified by, and adjusted for, the same variables as were used in the single- and multi-pollutant models. Cumulatively, we found significant, positive associations with deaths from each of the following: nonaccidental causes, lung cancer, cardiometabolic diseases, diabetes, cardiovascular disease, and ischemic heart disease, but no significant associations with cerebrovascular, respiratory disease, or COPD. The strongest overall, cumulative risk estimate was for mortality from diabetes (HR = 1.180; 95% CI: 1.125, 1.236). Mutually adjusted HRs for the individual pollutants from the three-pollutant models show that, in general, associations with mean – 5th percentile increases in PM_2.5_ and O_3_ are weaker than those for NO_2_, and consequently, HRs for increases in all three pollutants (vs. no increase in any pollutant) are similar to the product of the HRs for increases in O_3_ and NO_2_, or increases in PM_2.5_ and NO_2_.

**Table 2:** Hazard ratios (95% CIs) for cumulative risk estimates from two- and three-pollutant models, stratified by age and sex, adjusted for personal*^a^* and contextual*^b^* covariates.

Cause of mortality	PM_2.5_ + O_3_	PM_2.5_ + NO_2_	O_3_ + NO_2_	PM_2.5_ + O_3_ + NO_2_
Nonaccidental	1.038 (1.032, 1.044)	1.070 (1.062, 1.078)	1.074 (1.065, 1.083)	1.075 (1.067, 1.084)
Trachea, bronchus, and lung cancers	1.023 (1.005, 1.042)	1.086 (1.060, 1.114)	1.073 (1.045, 1.100)	1.078 (1.050, 1.106)
Cardiometabolic diseases	1.048 (1.038, 1.057)	1.062 (1.049, 1.076)	1.075 (1.061, 1.089)	1.075 (1.061, 1.089)
Diabetes	1.177 (1.139, 1.217)	1.145 (1.094, 1.198)	1.170 (1.117, 1.226)	1.180 (1.125, 1.236)
Cardiovascular	1.038 (1.028, 1.049)	1.057 (1.042, 1.071)	1.068 (1.054, 1.084)	1.068 (1.053, 1.083)
Ischemic heart disease	1.100 (1.086, 1.114)	1.118 (1.099, 1.137)	1.133 (1.113, 1.153)	1.137 (1.116, 1.157)
Cerebrovascular	0.964 (0.943, 0.986)	0.974 (0.945, 1.004)	0.988 (0.957, 1.019)	0.980 (0.949, 1.011)
Diseases of the respiratory system	0.968 (0.949, 0.988)	1.010 (0.983, 1.038)	1.008 (0.980, 1.036)	1.005 (0.977, 1.034)
COPD and allied conditions	0.979 (0.953, 1.006)	1.049 (1.012, 1.088)	1.037 (0.999, 1.076)	1.038 (0.999, 1.077)
Hazard ratios are per mean – 5th percentile (i.e., 5.0-μg/m^3^, 9.5-ppb, and 8.1-ppb increases in PM_2.5_, O_3_, and NO_2_, respectively).^***a***^Aboriginal ancestry, visible minority status, highest level of education, employment status, occupational class, immigrant status, marital status, income quintile. ^*b*^Census division and census tract–census division percent of immigrants, percent of adults without high school diploma, percent of subjects in lowest income quintile.				

*Results of additional analyses.* Analyses of effect modification are presented in Supplemental Material, Tables S2 and S3, for all nonaccidental mortality and cardiometabolic mortality, respectively. Among men, we found decreasing associations between exposure and risk of mortality among older subjects in all cases except with cardiometabolic deaths and NO_2_. Among women, however, we found this association only in the case of O_3_ (with both outcomes). Compared with those in the highest income quintile, subjects in the lowest had stronger associations with all three pollutants and nonaccidental mortality, but only with NO_2_ in the case of cardiometabolic mortality. Last, we found positive associations among Canadian-born subjects in all cases, but no associations among immigrants and exposure to PM_2.5_ or O_3_.

Our models in which we assigned exposure only at baseline were based on a slightly different set of subjects from those included in our main models. The baseline-exposure cohort consists exclusively of subjects for whom we had a postal code in 1991, regardless of availability in other years. This cohort had an age distribution and proportion of immigrants similar to those of our main cohort (results not shown). Here, with O_3_ we found associations with nonaccidental mortality nearly identical to those reported in our main models (i.e., 1.034, 95% CI: 1.028, 1.039); with PM_2.5_ we found similar, but attenuated associations (i.e., 1.025; 95% CI: 1.020, 1.031); and with NO_2_, we found notably weaker evidence of an association (i.e., 1.017; 95% CI: 1.009, 1.025).

Indirect adjustment for smoking behavior and obesity had very little impact on the HRs (generally in the range of 1–2% increase or decrease, depending on cause of death; the indirectly adjusted HRs presented in Supplemental Material, Table S4, can be compared with the non-indirectly adjusted HRs presented in Table 1).

In the case of all three pollutants, models using natural splines improved model fit compared to those that assumed linearity. We examined the spline plots for nonaccidental mortality and identified different shapes in the response curves for each pollutant (see Supplemental Material, Figure S3A–C). The natural spline fit for both PM_2.5_ and NO_2_ are supralinear, whereas the fit for O_3_ appears to be sublinear. Fits for all three pollutants were superior to their linear counterparts (*p* < 0.0001). Supralinear concentration–mortality associations are characterized by larger changes in risk for low concentrations compared with higher values, whereas sublinear associations have the opposite property (Pope et al. 2015). Estimates of excess deaths attributable to changes in air quality will thus depend on the exposure distribution, the size of population exposed to any concentration, and the shape of the concentration–mortality association. For PM_2.5_ and NO_2_, marginal changes in exposure in areas of low pollution will translate into larger marginal reductions in deaths compared with equivalent marginal changes in areas of higher pollution. For O_3_, the opposite pattern of excess deaths is predicted by our results. This suggests that strategies to improve air quality could be designed with regional differences for each pollutant to maximize improvement in population health.

## Discussion

We found positive associations between several common causes of death and exposure to generally low concentrations of PM_2.5_, O_3_, and NO_2_ in a nationally representative cohort of > 2.5 million Canadian adults who were followed for 16 years. The findings here corroborate and expand upon those reported in our earlier analysis (Crouse et al. 2012) with the previous cohort where we considered associations only with PM_2.5_ and 10 years of follow-up. The present study is among the first cohort studies to show associations between ambient concentrations of O_3_ and risk of mortality from several important causes of death, including cardiometabolic diseases, diabetes, cardiovascular disease, and ischemic heart disease, after controlling for both PM_2.5_ and NO_2_ together. Substantial spatial correlation between PM_2.5_ and O_3_ concentrations, however, makes it statistically difficult to separate the contributions to risk of the individual pollutants.

The patterns that we observed in our cumulative risk models for nonaccidental mortality (Table 2) are very similar to those reported by Jerrett et al. (2013) in their study of subjects across California using the same three pollutants, wherein they concluded that “a combination of NO_2_ and O_3_ is sufficient to characterize the toxicity of the pollutant mixture in this study, at least with respect to the three pollutants considered.”

Several previous cohort studies found stronger associations with PM_2.5_ and mortality from circulatory and cardiovascular disease than with nonaccidental causes (Cesaroni et al. 2013; Crouse et al. 2012; Jerrett et al. 2013; Lepeule et al. 2012). Here, however, we found the opposite results in our single- and multi-pollutant models. In fact, in our models adjusted for all three pollutants, we found no significant associations with PM_2.5_ and mortality from either cardiometabolic diseases (i.e., HR = 0.998; 95% CI: 0.983, 1.014) or cardiovascular disease (i.e., HR = 0.993; 95% CI: 0.977, 1.010). This may be attributable to collinearity or to the spatial nature of O_3_ and NO_2_, with O_3_ capturing regional variation in air pollution and NO_2_ capturing local scale variation (and PM_2.5_ capturing some of both local and regional variation). That is, our estimates of O_3_ could be acting as a better indicator of regional pollution (and potentially serving as a marker for secondary pollutants) than are our estimates of PM_2.5_, whereas NO_2_ is potentially serving as a better marker for relatively fresh combustion emissions, especially traffic. Therefore, including O_3_ and NO_2_ may be explaining these scales of spatial variation as they related to spatial patterns of mortality more effectively than PM_2.5_ alone. The results of the multi-pollutant models should, therefore, be interpreted with caution in regards to inferring potential causal pollutants.

As noted above, we found no positive associations between O_3_ and deaths from respiratory disease or COPD in any of our models. Jerrett et al. (2009a), in their large cohort study of nearly 450,000 subjects across the United States, observed positive associations between O_3_ and deaths from respiratory disease, in both single-pollutant models and in models adjusted also for PM_2.5_. They reported, however, that local temperature significantly modified the association, with no positive associations among subjects living in areas with long-term average daily maximum temperatures below 25.4°C. Given Canada’s typically cooler climate compared with most of the United States, this may explain in part why we found no associations between O_3_ and respiratory mortality.

We also found positive associations with NO_2_ and mortality from lung cancer and respiratory diseases. These results corroborate the findings of a previous Canadian population-based case–control study (Hystad et al. 2013) as well as the results of a recent meta-analysis on NO_2_ and lung cancer risk (Hamra et al. 2015). Very few cohort studies have considered associations between lung cancer or respiratory-related mortality and long-term exposure to NO_2_, and among those that have, the findings with respiratory mortality have been inconsistent. For example, a recent, national English cohort study (Carey et al. 2013) found stronger associations with NO_2_ and both lung cancer and respiratory mortality than with circulatory mortality. In California, however, Jerrett et al. (2013) found positive associations between NO_2_ and lung cancer, but found no significant associations with NO_2_ and respiratory mortality in either single- or joint-pollutant models with O_3_ and PM_2.5_.

The estimated associations with nonaccidental mortality and both PM_2.5_ and O_3_ were stronger in males than in females, nonimmigrants compared with immigrants, and among those in the lowest versus highest income quintile. Associations with nonaccidental mortality and NO_2_ were also stronger among those in the lowest versus highest income quintile. We found evidence of effect modification by age with PM_2.5_ (among men), O_3_ (among both men and women), and NO_2_ (among men). There have been few, and mixed, reports on effect modification by age in the association between air pollution and mortality. In their study of NO_2_ and mortality in three Canadian cities, Chen et al. (2013) found no evidence of effect modification by age, but they did not stratify by sex. Conversely, Cesaroni et al. (2013) reported stronger associations between NO_2_ and mortality from nonaccidental causes among younger subjects in their large Italian cohort. It is also somewhat perplexing that for PM_2.5_ and O_3_ we found stronger associations with mortality among nonimmigrants, but for NO_2_, associations were stronger among immigrants. Part of this pattern could be explained by the fact that immigrants tend to be healthier than nonimmigrant Canadians (Ng 2011) and that they tend to congregate in larger cities where concentrations of NO_2_ are generally higher. It is challenging to study and to understand health outcomes among Canadian immigrants broadly, because there is substantial heterogeneity in health outcomes within immigrant subgroups by birthplace, by area of residence in Canada, and by period of immigration (Ng 2011). The role of age and immigrant status in modifying associations between individual pollutants and mortality merits further investigation.

Most of our other findings are generally consistent with those reported elsewhere. For example, we found essentially no associations with cerebrovascular mortality in any of our models. A recent meta-analysis on long-term exposures to ambient air pollution and all-cause mortality (Hoek et al. 2013) reported pooled HRs (per 10 μg/m^3^) based on cohort studies conducted around the world of 1.06 (95% CI: 1.04, 1.08) with PM_2.5_ and of 1.05 (95% CI: 1.03, 1.08) with NO_2_. In our comparable models for nonaccidental mortality—and calculated per increment in exposure of 10 μg/m^3^—we found HR = 1.07 (95% CI: 1.06, 1.08) with PM_2.5_ and HR = 1.03 (95% CI: 1.03, 1.04) with NO_2_; both of which fit within the ranges reported in that meta-analysis. As we have outlined earlier, there are many differences between the study design of this and our previous CanCHEC analysis, including the addition of immigrants. Here we found strong effect modification by immigrant status, and in models for nonaccidental mortality excluding immigrants, we found HR per 10 μg/m^3^ = 1.10 (95% CI: 1.08, 1.11), which is very close to that reported in the most comparable model from our earlier study (HR = 1.11; 95% CI: 1.10, 1.12).

A key strength of this study is the large size of this population-based cohort, as well as the fact that we included subjects from all across Canada—including those in rural and remote, northern locations. We were able to adjust directly and indirectly for many individual and contextual risk factors for mortality, although we did lack data on smoking, obesity, physical activity, and alcohol consumption, among other potential confounders. Similar to what has been reported elsewhere (Chen et al. 2013; Villeneuve et al. 2013), indirect adjustment for smoking behaviour and obesity had very little impact on the HRs (generally in the range of 1–2% increase or decrease, depending on cause of death).

Another key strength of this study is that we were able to assign exposures to the representative point corresponding to the 6-digit residential postal code of each subject for each year of follow-up (and up to 7 years preceding follow-up). This approach allowed us to reduce exposure misclassification bias that could arise when subjects move between different pollution environments given our estimates of long-term moving windows of exposure that took mobility patterns into consideration. We acknowledge, however, that people do not spend all of their time at their residence; on a daily basis they may commute to school or work or spend parts of their day in other areas of the city. As such, our exposure estimates cannot reflect subjects’ complete daily or long-term exposures.

Although we had residential location information for each year of follow-up, and we were able to recalculate the contextual covariates each census year, all personal covariates were available only at baseline. A related limitation of our analysis is that our estimates of exposure were based on fixed periods of time, and may not accurately describe long-term changes in pollutant concentrations over time. We also emphasize that our pollutant models were developed independently, with different predictor variables, and represent different time periods, leading to varying abilities to adequately describe the true pollutant patterns and concentrations.

The results of our baseline-exposure analyses suggest that the benefit achieved (i.e., reduction in exposure misclassification) through tracking subjects’ residential mobility patterns and assigning time-varying exposure estimates is related directly to the spatial resolution and spatial variability of the exposure estimates. In the case of O_3_, for which our exposure estimates were based on a very broad 21-km spatial resolution, we found little difference between the results of our no-mobility and main cohort survival models. This finding also suggests that not enough people in our main cohort were moving to or from areas characterized by substantially different levels of O_3_ to produce HRs substantially different from those produced in models that assumed that the baseline exposure represented long-term exposures. In the case of NO_2_, however, the weaker associations in models not incorporating mobility are consistent with finer spatial variation captured by our NO_2_ models. This suggests that mobility within a small area such as a city may be important for fine-scale exposure models but not as important for those models defined at a broader spatial scale.

As shown in our concentration–response plots, associations with nonaccidental mortality increase monotonically throughout the concentration range, as evidenced by the natural spline fit.

## Conclusions

Our results provide evidence that long-term exposures to three key components of ambient air pollution are associated with increased risk of nonaccidental and cause-specific mortality. Our cumulative risk models suggest that exposure to PM_2.5_ alone does not fully characterize the toxicity of the atmospheric mix or fully explain the risk of mortality associated with exposure to ambient pollution. Correlations between the pollutants do, however, make it challenging to tease out the independent contributions to risk of mortality of each pollutant. Our cumulative risk estimates, however, describe associations with the overall mixture of pollutants more effectively than do the estimates from the multiple-pollutant models. These observations suggest that efforts should be made to model the toxicity of atmospheric mixtures when modeling population burden of disease attributable to air pollution exposure.

## Supplemental Material

(1.6 MB) PDF
