# Supplementary material for: Ambient PM2.5, O3, and NO2 Exposures and Associations with Mortality over 16 Years of Follow-Up in the Canadian Census Health and Environment Cohort (CanCHEC)
Source: Environ Health Perspect. 2015 Nov 1;123(11):1180–6. doi: 10.1289/ehp.1409276 (PMC4629747; doi:10.1289/ehp.1409276)
Supplement: (1.6 MB) PDF [file ehp.1409276.s001.acco.pdf]

Environ Health Perspect
DOI: 10.1289/ehp.1409276

Note to Readers: *EHP* strives to ensure that all journal content is accessible to all readers. However, some figures and Supplemental Material published in *EHP* articles may not conform to 508 standards due to the complexity of the information being presented. If you need assistance accessing journal content, please contact ehp508@niehs.nih.gov. Our staff will work with you to assess and meet your accessibility needs within 3 working days.

Supplemental Material

Ambient $PM_{2.5}$, O_3, and NO_2 Exposures and Associations with Mortality over 16 Years of Follow-Up in the Canadian Census Health and Environment Cohort (CanCHEC)

Dan L. Crouse, Paul A. Peters, Perry Hystad, Jeffrey R. Brook, Aaron van Donkelaar, Randall V. Martin, Paul J. Villeneuve, Michael Jerrett, Mark S. Goldberg, C. Arden Pope III, Michael Brauer, Robert D. Brook, Alain Robichaud, Richard Menard, and Richard T. Burnett

Table of Contents

Table S1. Descriptive statistics of cohort subjects at baseline and fully-adjusted hazard ratios for risk factors included in the survival models for all non-accidental causes of death (and with each pollutant individually).

Table S2. Associations between mortality from non-accidental deaths and $PM_{2.5}$, O_3, and NO_2, and effect modification by selected characteristics. All models stratified by age and sex, adjusted for personal and contextual covariates; hazard ratios for $PM_{2.5}$ per 10 $\mu g/m^3$, hazard ratios for O_3 and NO_2 per 10 ppb.

Table S3. Associations between mortality from cardio-metabolic diseases and $PM_{2.5}$, O_3, and NO_2, and effect modification by selected characteristics. All models stratified by age and sex, adjusted for personal and contextual covariates; hazard ratios for $PM_{2.5}$ per 10 $\mu g/m^3$, hazard ratios for O_3 and NO_2 per 10 ppb.

Table S4. Hazard ratios (and 95% confidence intervals) for mortality by pollutant in single- and multi-pollutant models: models stratified by age and sex, adjusted for personal and contextual

covariates; indirectly adjusted for smoking and obesity; hazard ratios per mean-5th percentile (i.e., 5.0 μg/m^3, 9.5 ppb, and 8.1 ppb increases in $PM_{2.5}$, O_3, and NO_2, respectively).

Figure S1. Comparison of satellite-derived estimates of $PM_{2.5}$ (median 1998-2006) with observations from fixed-site stations (mean 1984-2006) in 10 Canadian cities.

Figure S2. Maps of exposures as assigned to subjects; insets for Vancouver, Toronto, and Montreal.

Figure S3. Concentration response plots for mortality by pollutant in single-pollutant models: models stratified by age and sex, adjusted for personal and contextual covariates. a) $PM_{2.5}$ (mean: 8.9 μg/m^3; knots: 3.9, 8.6, 14.4 μg/m^3). b) O_3 (mean: 39.6 ppb; knots: 30.0, 38.9, 50.7 ppb). c) NO_2 (mean: 11.6 ppb; knots: 3.1, 9.8, 23.4 ppb).

Table S1: Descriptive statistics of cohort subjects at baseline and fully-adjusted hazard ratios for risk factors included in the survival models for all non-accidental causes of death (and with each pollutant individually).

Subject characteristics at baseline	Subjects n[a] (%)	Deaths n[a] (%)	PM_{25} mean ± SD ($\mu g/m^3$)	O_3 mean ± SD (ppb)	NO_2 mean ± SD (ppb)
All	2,521,525 (100)	301,115 (100)	8.9 ± 3.4	39.6 ± 6.6	11.6 ± 6.7
Sex					
men	1,254,800 (49.8)	184,055 (61.1)	8.9 ± 3.4	39.5 ± 6.6	11.5 ± 6.7
women	1,266,725 (50.2)	117,060 (38.9)	9.0 ± 3.4	39.6 ± 6.6	11.8 ± 6.7
Age					
25-34	709,075 (28.1)	6,265 (2.1)	8.9 ± 3.3	39.4 ± 6.3	11.7 ± 6.6
35-64	1,434,235 (56.9)	119,840 (39.8)	8.9 ± 3.4	39.6 ± 6.6	11.5 ± 6.7
65-74	251,845 (10.0)	112,995 (37.5)	9.0 ± 3.5	39.8 ± 6.9	12.0 ± 7.0
75-90	126,370 (5.0)	62,015 (20.6)	8.9 ± 3.6	39.7 ± 6.9	12.2 ± 7.1
Aboriginal Ancestry					
yes	100,665 (4.0)	7,455 (2.5)	7.6 ± 2.5	36.4 ± 5.4	8.1 ± 5.6
no	2,420,860 (96.0)	293,660 (97.5)	9.0 ± 3.4	39.7 ± 6.6	11.8 ± 6.7
Visible minority status[b]					
yes	163,820 (6.5)	9,355 (3.1)	10.1 ± 3.7	40.4 ± 6.2	17.9 ± 5.9
no	2,357,705 (93.5)	291,760 (96.9)	8.8 ± 3.4	39.5 ± 6.6	11.2 ± 6.6
Marital status					
divorced/separate/widowed	328,230 (13.0)	74,970 (24.9)	9.0 ± 3.4	39.6 ± 6.6	12.4 ± 7.0
single	306,915 (12.2)	25,955 (8.6)	9.1 ± 3.4	39.4 ± 6.1	13.4 ± 7.3
married/common law	1,886,380 (74.8)	200,190 (66.5)	8.9 ± 3.4	39.6 ± 3.7	11.2 ± 6.5
Income quintiles					
lowest	405,105 (16.1)	72,165 (24.0)	8.9 ± 3.4	39.4 ± 6.5	12.2 ± 7.3
lower middle	485,370 (19.2)	74,925 (24.9)	9.0 ± 3.4	39.6 ± 6.6	12.0 ± 7.0
middle	528,840 (21.0)	56,350 (18.7)	8.9 ± 3.4	39.5 ± 6.6	11.6 ± 6.7
upper middle	547,375 (21.7)	49,325 (16.4)	8.9 ± 3.4	39.6 ± 6.6	11.4 ± 6.5
upper	554,835 (22.0)	48,350 (16.1)	8.9 ± 3.4	39.6 ± 6.6	11.2 ± 6.3
Education					
< high school graduation	867,110 (34.4)	171,755 (57.0)	8.8 ± 3.4	39.3 ± 6.7	11.0 ± 7.1
high school graduation with or without trade certificate	924,200 (36.7)	85,000 (28.2)	8.9 ± 3.4	39.5 ± 6.6	11.4 ± 6.5
some postsecondary, or college diploma	393,955 (15.6)	25,160 (8.4)	8.9 ± 3.4	39.7 ± 6.6	11.9 ± 6.4
≥ university degree	336,260 (13.3)	19,200 (6.4)	9.3 ± 3.4	40.1 ± 6.2	13.5 ± 6.4

Occupational class					
not applicable	601,130 (23.8)	188,760 (62.7)	8.9 ± 3.5	39.5 ± 6.7	11.7 ± 7.1
unskilled	196,550 (7.8)	15,275 (5.1)	8.8 ± 3.4	39.3 ± 6.7	11.3 ± 7.0
semi-skilled	617,035 (24.5)	35,080 (11.7)	9.0 ± 3.4	39.7 ± 6.6	11.7 ± 6.7
technical	610,375 (24.2)	37,910 (12.6)	8.8 ± 3.4	39.4 ± 6.6	11.1 ± 6.5
management	209,370 (8.3)	12,515 (4.2)	9.1 ± 3.3	39.9 ± 6.5	12.2 ± 6.3
professional	287,065 (11.4)	11,575 (3.8)	9.1 ± 3.4	39.8 ± 6.4	12.5 ± 6.4
Labour force status					
not in labour force	697,590 (27.7)	203,970 (67.7)	8.9 ± 3.5	39.4 ± 6.7	11.7 ± 7.0
unemployed	171,095 (6.8)	9,700 (3.2)	8.7 ± 3.3	38.9 ± 6.2	11.3 ± 7.2
employed	1,652,840 (65.5)	87,445 (29.0)	9.0 ± 3.4	39.7 ± 6.6	11.7 ± 6.6
Immigrant status					
Canadian born	2,035,550 (80.7)	240,525 (79.9)	8.6 ± 3.3	39.1 ± 6.5	10.7 ± 6.4
immigrant	485,975 (19.3)	60,590 (20.1)	10.1 ± 3.7	41.3 ± 6.7	15.8 ± 6.6

[a.] subject counts rounded to nearest 5; percentages based on original values

[b.] Visible minorities are persons (other than aboriginal persons), who are non-Caucasian in race or non-white in colour.

Table S2. Associations between mortality from non-accidental deaths and $PM_{2.5}$, O_3, and NO_2, and effect modification by selected characteristics. All models stratified by age and sex, adjusted for personal[a] and contextual[b] covariates; hazard ratios for $PM_{2.5}$ per 10 $\mu g/m^3$, hazard ratios for O_3 and NO_2 per 10 ppb.

**Effect modifier**	**Events n (%)**	**$PM_{2.5}$ HR (95% CI)**	**P[c]**	**O_3 HR (95% CI)**	**P[c]**	**NO_2 HR (95% CI)**	**P[c]**
None	301,115 (100)	1.072 (1.060, 1.084)		1.033 (1.027, 1.038)		1.065 (1.056, 1.074)	
Women by age during follow-up (years)							
all ages	117,060 (38.9)	1.035 (1.017, 1.054)		1.014 (1.005, 1.023)		1.065 (1.051, 1.079)	
less than 60	17,360 (5.8)	1.061 (1.011, 1.113)		1.028 (1.004, 1.053)		1.175 (1.134, 1.218)	
60-69	17,875 (5.9)	1.048 (1.000, 1.098)		1.032 (1.009, 1.056)		1.047 (1.012, 1.085)	
70-79	34,655 (11.5)	1.041 (1.007, 1.075)		1.021 (1.005, 1.038)		1.094 (1.067, 1.121)	
80 - 89	47,170 (15.7)	1.021 (0.994, 1.050)		0.996 (0.982, 1.009)		1.018 (0.997, 1.039)	
			0.33		0.00		0.45
Men, by age during follow-up (years)							
all ages	184,055 (61.1)	1.097 (1.081, 1.113)		1.044 (1.037, 1.052)		1.065 (1.054, 1.077)	
less than 60	25,670 (8.5)	1.137 (1.093, 1.183)		1.073 (1.052, 1.094)		1.134 (1.101, 1.168)	
60-69	37,690 (12.5)	1.121 (1.085, 1.157)		1.062 (1.045, 1.079)		1.070 (1.044, 1.096)	
70-79	65,535 (21.8)	1.097 (1.071, 1.124)		1.039 (1.026, 1.051)		1.050 (1.031, 1.070)	
80 - 89	55,160 (18.3)	1.065 (1.038, 1.093)		1.025 (1.012, 1.038)		1.040 (1.020, 1.061)	
			0.02		0.00		0.00
Income							
Bottom quintile	72,165 (24.0)	1.099 (1.074, 1.124)		1.044 (1.033, 1.056)		1.149 (1.130, 1.168)	
Top quintile	48,350 (16.1)	1.049 (1.020, 1.079)		1.021 (1.007, 1.035)		0.987 (0.965, 1.009)	
			0.01		0.01		0.00
Immigrant status							
Non-immigrant	240,525 (79.9)	1.095 (1.081, 1.109)		1.043 (1.036, 1.049)		1.064 (1.054, 1.074)	
Immigrant	60,590 (20.1)	1.001 (0.978, 1.024)		0.996 (0.985, 1.008)		1.073 (1.053, 1.093)	
			0.00		0.00		0.43

[a.] personal covariates: aboriginal ancestry, visible minority status, highest level of education, employment status, occupational class, immigrant status, marital status, income quintile

[b.] contextual covariates: census division and census tract – census division % of immigrants, % of adults without high school diploma, % of subjects in lowest income quintile

[c.] P-value for Q-statistic (test for effect modification)

Table S3. Associations between mortality from cardio-metabolic diseases and $PM_{2.5}$, O_3, and NO_2, and effect modification by selected characteristics. All models stratified by age and sex, adjusted for personal[a] and contextual[b] covariates; hazard ratios for $PM_{2.5}$ per 10 $\mu g/m^3$, hazard ratios for O_3 and NO_2 per 10 ppb.

Effect modifier	Events n (%)	$PM_{2.5}$ HR (95% CI)	P[c]	O_3 HR (95% CI)	P[c]	NO_2 HR (95% CI)	P[c]
None	117,495 (100)	1.079 (1.060, 1.098)		1.048 (1.039, 1.057)		1.050 (1.036, 1.064)	
Women by age during follow-up (years)							
all ages	43,210 (36.8)	1.065 (1.034, 1.097)		1.040 (1.025, 1.055)		1.051 (1.028, 1.075)	
less than 60	3,145 (2.7)	1.158 (1.032, 1.299)		1.101 (1.040, 1.165)		1.226 (1.128, 1.332)	
60-69	4,635 (3.9)	1.090 (0.994, 1.196)		1.074 (1.027, 1.124)		1.047 (0.977, 1.121)	
70-79	12,910 (11.0)	1.071 (1.016, 1.130)		1.056 (1.029, 1.085)		1.070 (1.027, 1.114)	
80 - 89	22,520 (19.2)	1.049 (1.008, 1.092)		1.015 (0.995, 1.036)		1.023 (0.992, 1.054)	
			0.41		0.01		0.37
Men, by age during follow-up (years)							
all ages	74,285 (63.2)	1.088 (1.064, 1.113)		1.052 (1.041, 1.064)		1.051 (1.033, 1.069)	
less than 60	9,095 (7.7)	1.133 (1.059, 1.211)		1.084 (1.049, 1.121)		1.036 (0.985, 1.090)	
60-69	13,930 (11.9)	1.127 (1.069, 1.189)		1.076 (1.048, 1.105)		1.066 (1.024, 1.110)	
70-79	26,730 (22.7)	1.114 (1.073, 1.157)		1.054 (1.035, 1.074)		1.056 (1.026, 1.087)	
80 - 89	24,530 (20.9)	1.029 (0.990, 1.069)		1.025 (1.006, 1.044)		1.037 (1.007, 1.068)	
			0.01		0.00		0.65
Income							
Bottom quintile	30,460 (25.9)	1.099 (1.061, 1.139)		1.053 (1.035, 1.072)		1.134 (1.105, 1.163)	
Top quintile	17,145 (14.6)	1.097 (1.047, 1.149)		1.053 (1.030, 1.077)		0.964 (0.929, 1.001)	
			0.95		1.00		0.00
Immigrant status							
Non-immigrant	93,365 (79.5)	1.114 (1.091, 1.137)		1.067 (1.056, 1.078)		1.042 (1.026, 1.058)	
Immigrant	24,130 (20.5)	0.977 (0.942, 1.014)		0.983 (0.965, 1.002)		1.080 (1.048, 1.112)	
			0.00		0.00		0.04

[a.] personal covariates: aboriginal ancestry, visible minority status, highest level of education, employment status, occupational class, immigrant status, marital status, income quintile

[b.] contextual covariates: census division and census tract – census division % of immigrants, % of adults without high school diploma, % of subjects in lowest income quintile

[c.] P-value for Q-statistic (test for effect modification)

Table S4. Hazard ratios (and 95% confidence intervals) for mortality by pollutant in single- and multi-pollutant models: models stratified by age and sex, adjusted for personal[a] and contextual[b] covariates; indirectly adjusted for smoking and obesity; hazard ratios per mean-5th percentile (i.e., 5.0 μg/m^3, 9.5 ppb, and 8.1 ppb increases in $PM_{2.5}$, O_3, and NO_2, respectively).

	**Non-accidental**	**Trachea, bronchus, and lung cancers**	**Cardio-metabolic diseases**	**Diabetes**	**Cardiovascular**	**Ischemic heart disease**	**Cerebrovascular**	**Diseases of the respiratory system**	**Chronic obstructive pulmonary disease and allied conditions**
$PM_{2.5}$ alone	1.048 (1.040,1.056)	1.064 (1.040, 1.088)	1.047 (1.037, 1.057)	1.155 (1.118, 1.193)	1.038 (1.027, 1.049)	1.099 (1.084, 1.113)	0.969 (0.948, 0.991)	0.986 (0.967, 1.007)	1.008 (0.981, 1.036)
$PM_{2.5}$ adjusted for O_3 and NO_2	1.015 (1.002, 1.028)	1.059 (1.020, 1.100)	0.998 (0.983, 1.014)	1.052 (1.002, 1.105)	0.993 (0.977, 1.010)	1.029 (1.008, 1.051)	0.939 (0.908, 0.971)	0.984 (0.954, 1.016)	1.016 (0.973, 1.061)
O_3 alone	1.043 (1.036, 1.050)	1.036 (1.015, 1.057)	1.054 (1.045, 1.064)	1.163 (1.128, 1.199)	1.045 (1.035, 1.055)	1.100 (1.086, 1.113)	0.990 (0.970, 1.011)	0.984 (0.966, 1.003)	0.992 (0.966, 1.017)
O_3 adjusted for $PM_{2.5}$ and NO_2	1.028 (1.017, 1.039)	0.990 (0.958, 1.022)	1.052 (1.037, 1.066)	1.123 (1.074, 1.174)	1.046 (1.030, 1.061)	1.073 (1.053, 1.093)	1.033 (1.001, 1.065)	0.990 (0.962, 1.019)	0.974 (0.937, 1.012)
NO_2 alone	1.054 (1.044, 1.065)	1.061 (1.030, 1.093)	1.042 (1.029, 1.055)	1.04 (1.001, 1.085)	1.043 (1.029, 1.057)	1.065 (1.048, 1.083)	1.008 (0.981, 1.037)	1.032 (1.006, 1.058)	1.053 (1.017, 1.090)
NO_2 adjusted for $PM_{2.5}$ and O_3	1.044 (1.033, 1.056)	1.043 (1.019, 1.067)	1.034 (1.020, 1.047)	1.008 (0.967, 1.051)	1.037 (1.022, 1.051)	1.042 (1.024, 1.061	1.024 (0.995, 1.054)	1.039 (1.012, 1.067)	1.052 (1.015, 1.092)

[a.] aboriginal ancestry, visible minority status, highest level of education, employment status, occupational class, immigrant status, marital status, income quintile

[b.] census division and census tract-census division % of immigrants, % of adults without high school diploma, % of subjects in lowest income quintile

Figure S1. Comparison of satellite-derived estimates of $PM_{2.5}$ (median 1998-2006) with observations from fixed-site stations (mean 1984-2006) in 10 Canadian cities.

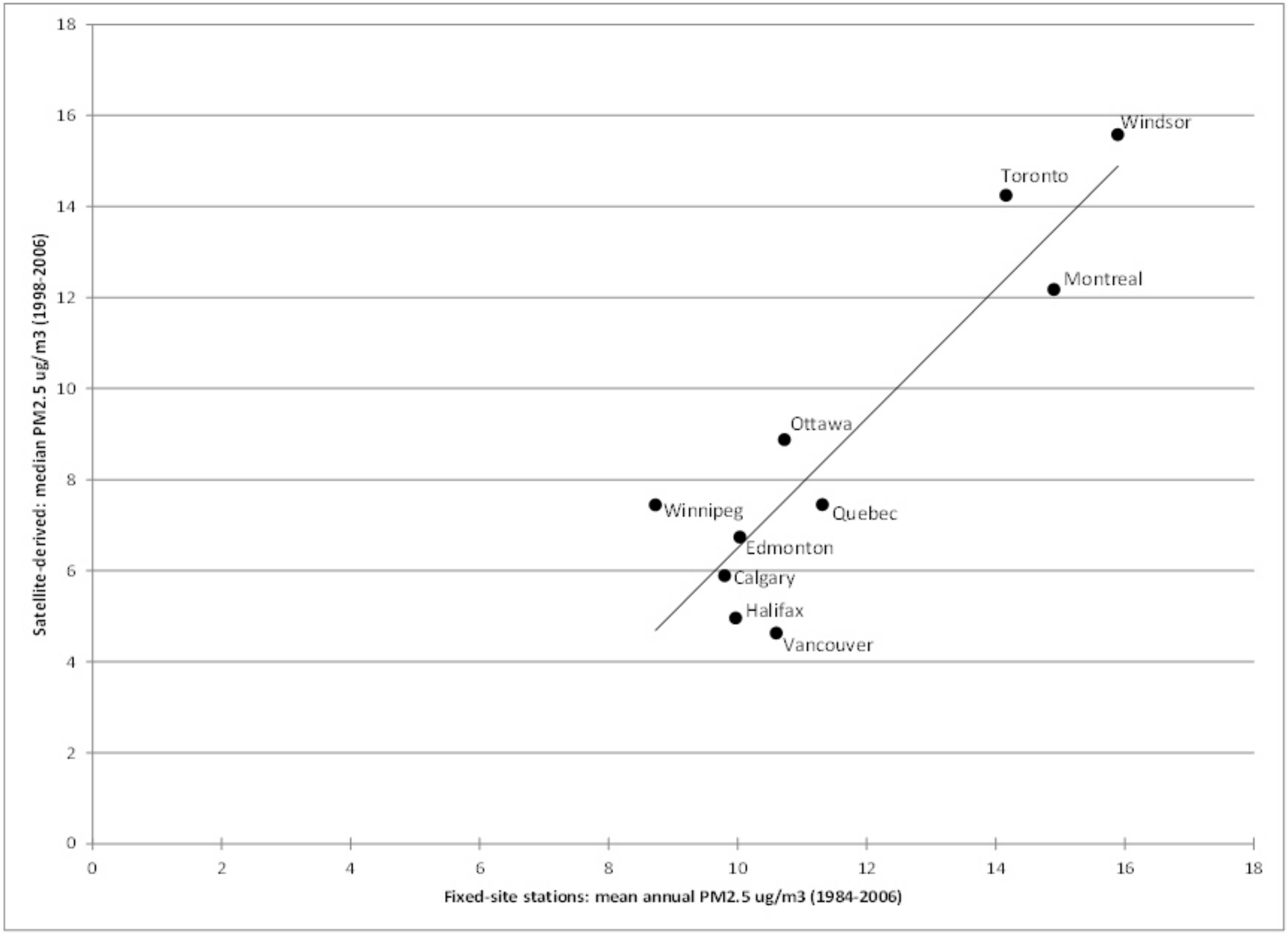

Figure S2. Maps of exposures as assigned to subjects; insets for Vancouver, Toronto, and Montreal.

Panel a) $PM_{2.5}$

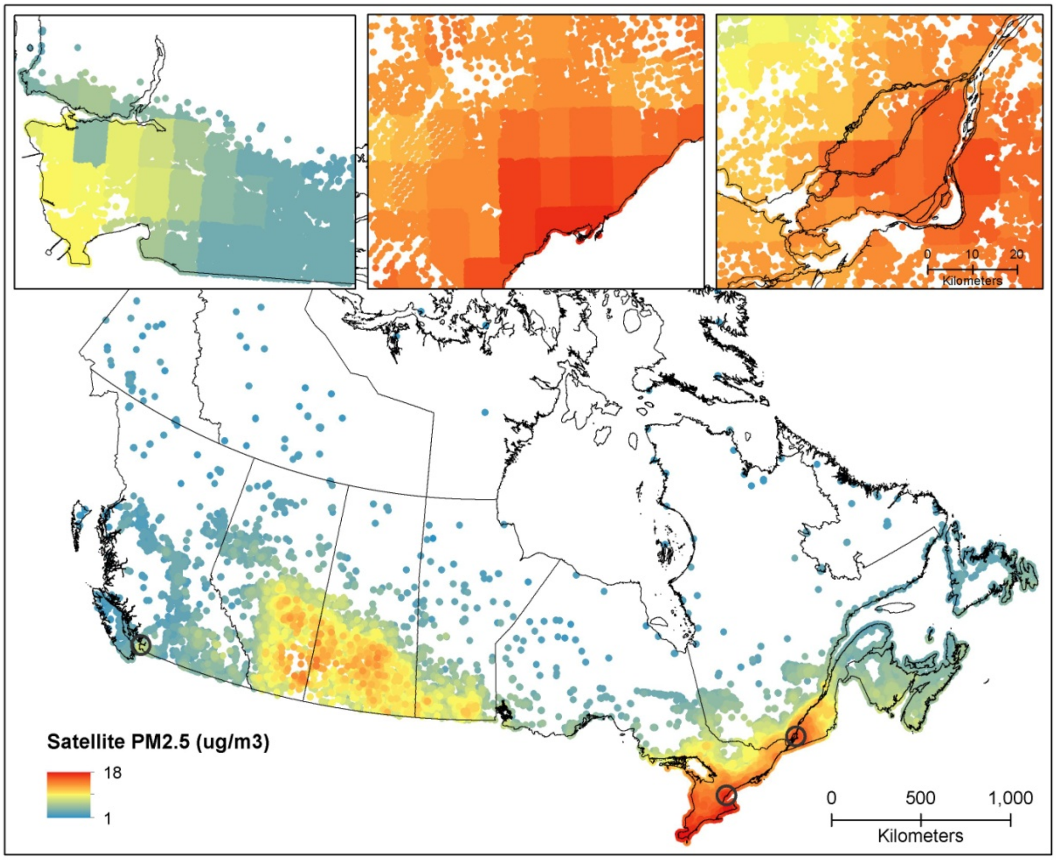

Panel b) O_3

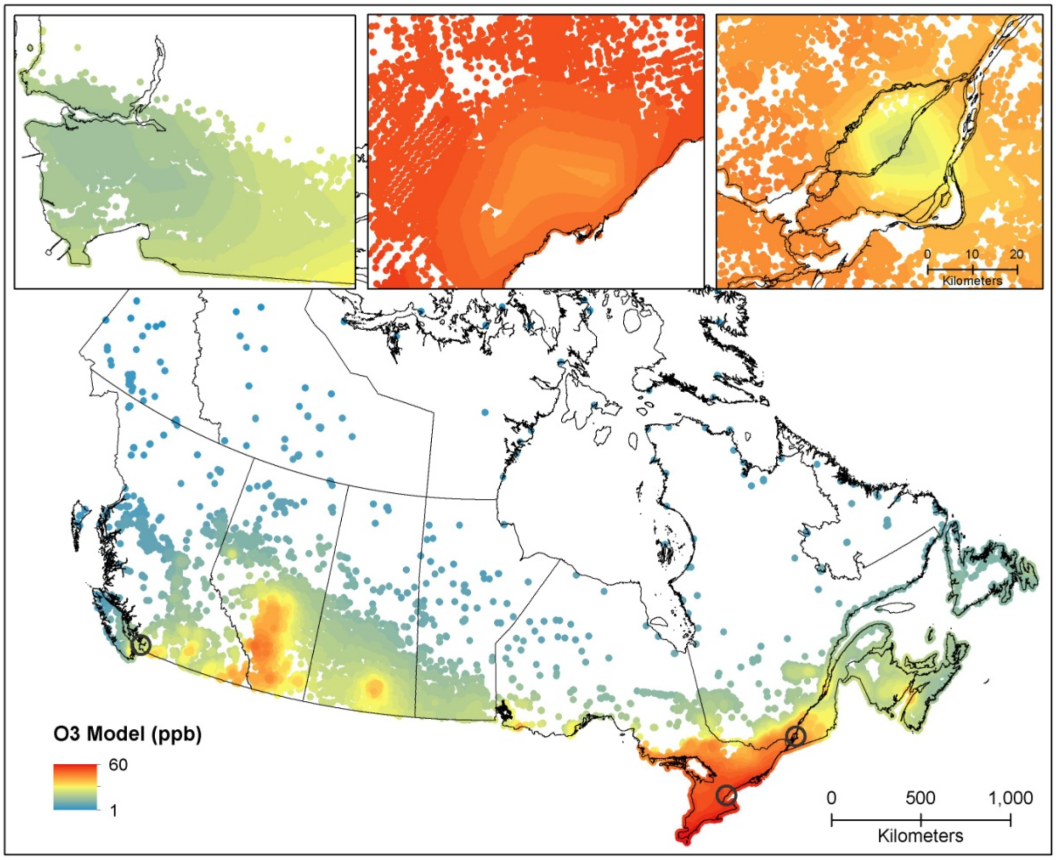

Panel c) NO_2

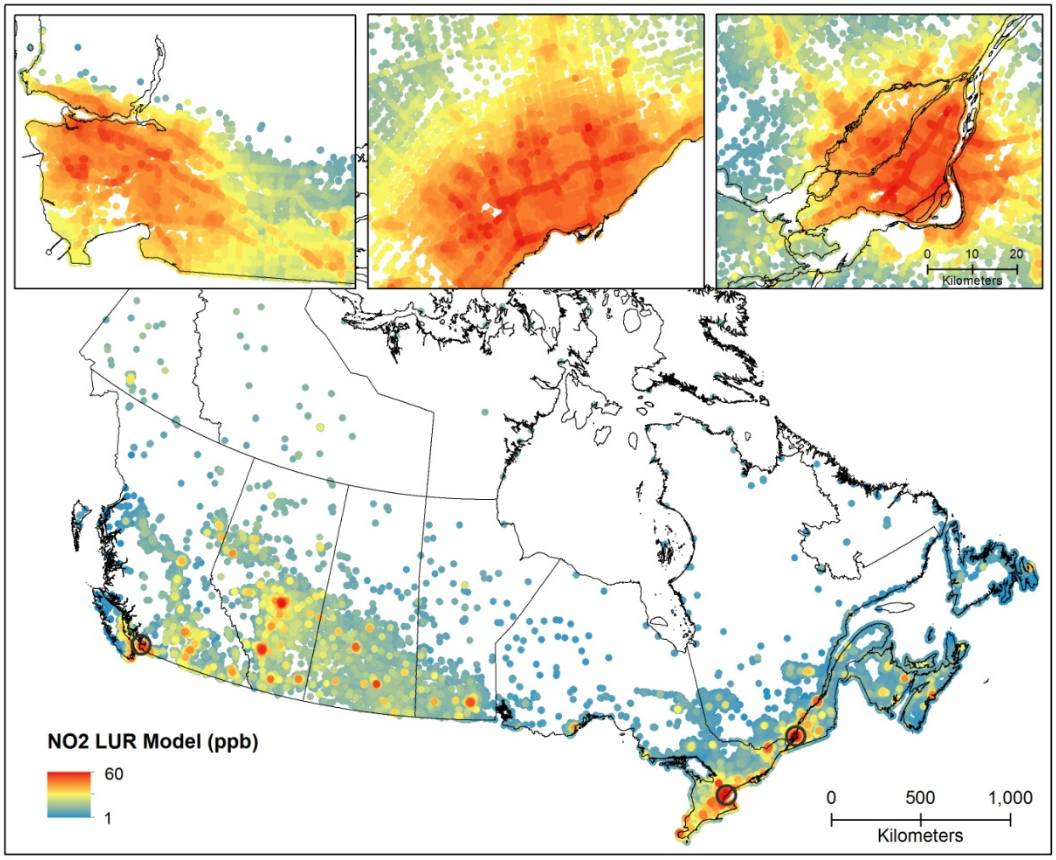
0 10 20
Kilometers
NO2 LUR Model (ppb)
60
1
0 500 1,000
Kilometers

Figure S3. Concentration response plots for mortality by pollutant in single-pollutant models: models stratified by age and sex, adjusted for personal[a] and contextual[b] covariates. a) $PM_{2.5}$ (mean: 8.9 μg/m^3; knots: 3.9, 8.6, 14.4 μg/m^3). b) O_3 (mean: 39.6 ppb; knots: 30.0, 38.9, 50.7 ppb). c) NO_2 (mean: 11.6 ppb; knots: 3.1, 9.8, 23.4 ppb).

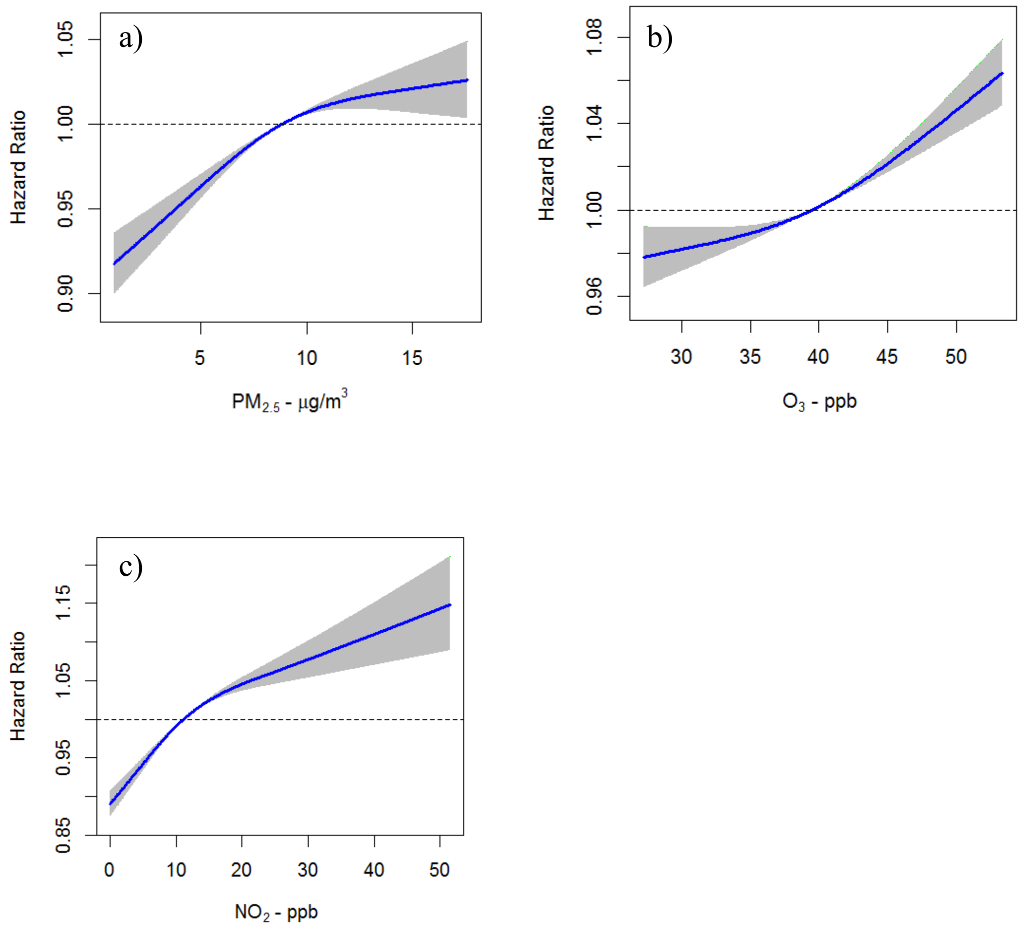

[a.] aboriginal ancestry, visible minority status, highest level of education, employment status, occupational class, immigrant status, marital status, income quintile

[b.] census division and census tract-census division % of immigrants, % of adults without high school diploma, % of subjects in lowest income quintile
